# Graphic Methods in Heart Disease

**Published:** 1909-12

**Authors:** 


**Graphic Methods in Heart Disease.** By JOHN HAY, M.D. Pp. xvii, 184. London: Oxford Medical Publications. 1909.

This is a very good little book. Dr. Hay has undertaken a difficult task. The study by graphic records of the derangements of the circulation caused by disease is a highly technical matter, and its exponent, if he wishes to be adequately understood, must be very lucid. But this exposition is not merely lucid. By a judicious blending of theory and illustration, Dr. Hay has succeeded in writing a book which may well interest all occupied in the practice of medicine, and not simply those who are specially interested in the study of heart disease. The more controversial parts of the subject have, we think wisely, been avoided, and the error, common among "polygraphers," of finding out too much from their tracings, is but little in evidence in these pages. The book is, therefore, eminently fitted to act as a guide to all who want an introduction to this most interesting branch of clinical medicine.

